# Rapid and Sensitive Chemical Analysis of Individual Picolitre Droplets by Mass Spectrometry

**DOI:** 10.1021/acs.analchem.4c05458

**Published:** 2024-12-24

**Authors:** Jim S. Walker, Bryan R. Bzdek

**Affiliations:** School of Chemistry, University of Bristol, Bristol BS8 1TS, United Kingdom

## Abstract

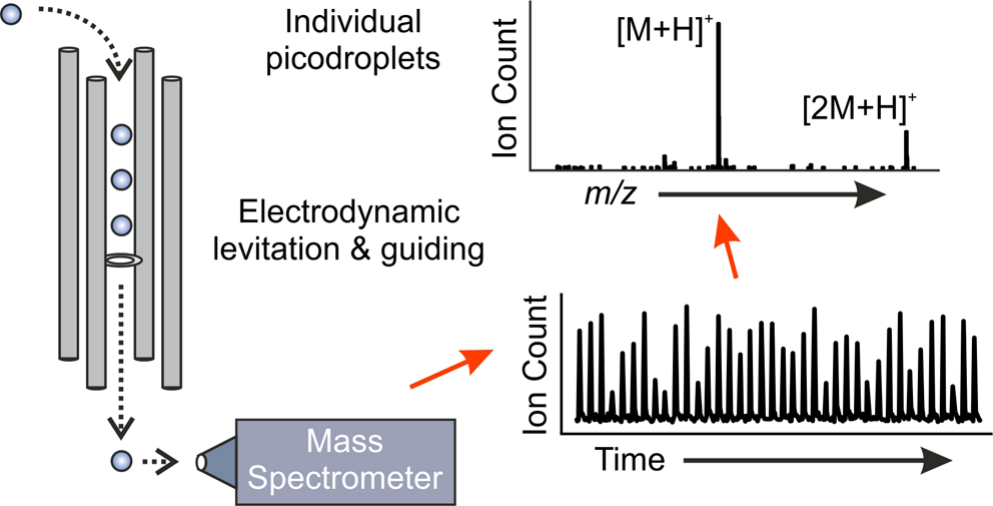

Aerosol droplets are unique microcompartments containing microscopic amounts of material and exhibiting surprising chemical reactivity. Although a diverse set of tools exists to characterize the chemical composition of individual submicron particles in air, comparatively fewer approaches can chemically analyze individual, airborne picolitre droplets. We describe a novel approach for mass spectrometric analysis of individual aqueous picolitre droplets (∼2–180 pL volume) containing down to ∼1 pg analyte mass per droplet. Individual droplets are generated using a microdroplet dispenser, imparted a small amount of net charge, and guided to the inlet of a high-resolution mass spectrometer using a linear quadrupole-electrodynamic balance. Analyte molecules within the aqueous droplet are ionized using droplet assisted ionization, where droplet breakup within the mass spectrometer inlet leads to generation of molecular ions. This single droplet mass spectrometry approach is demonstrated for small molecules and proteins. The approach generates clean mass spectra, permits timing of droplet delivery for chemical analysis, and, by avoiding a separate ionization stage, avoids potential artifacts arising from current electrospray-based approaches for picolitre droplet analysis. It is anticipated this approach will permit exploration of the factors governing accelerated chemical reactions in aerosol droplets and will be suitable for sensitive analysis of particularly precious samples in different application domains.

## Introduction

Microcompartments like aerosol droplets are central to diverse domains including atmospheric chemistry and climate change, human health and disease transmission, and materials synthesis. Aerosols are unique because they are containerless reaction vessels with high surface area-to-volume ratios and hold only microscopic amounts of matter.^1^ A surprising observation of recent years is that some chemical reactions in aerosols can be vastly accelerated (up to 10^7^ times) compared to the same reaction in macroscopic solutions.^2−6^ Explanations for observations of accelerated chemistry include high reactant concentrations, interfacial confinement of reactants and potential modifications of their Gibbs energies, and interfacial electric fields altering reaction pathways.^7−12^ However, many approaches to investigate accelerated chemical reactions in aerosol droplets have studied charged droplets generated by variations of electrospray ionization (ESI), including extractive ESI, desorption ESI, ESI/electrosonic spray, and paper spray ionization.^13−17^ In many applications, droplet formation and chemical analysis are tightly coupled. Moreover, these approaches can generate highly unconstrained and dynamic reaction conditions, with polydispersity in droplet size, rapid solvent evaporation altering reagent concentrations, and poor constraints on droplet charge.

A rich diversity of approaches to measure the chemical composition of single aerosol particles exists for nominally dry <10 μm diameter particles.^18−21^ In contrast, comparably fewer approaches exist for liquid droplets >1 μm diameter. Acoustic levitation has been coupled with mass spectrometry (MS) through ionization approaches including laser desorption/ionization,^22^ field induced droplet ionization,^23−25^ charge and matrix-assisted laser desorption/ionization,^26^ atmospheric pressure chemical ionization,^27^ secondary ESI,^28^ and direct analysis in real time.^29^ However, acoustic levitation generally is limited to microlitre sample volumes that may not accurately represent the unique environment of aerosol droplets. Electrodynamic levitation approaches for picolitre volume aerosol droplets have been coupled with MS through ionization approaches including paper spray,^30,31^ corona discharge,^32^ thermal desorption glow discharge,^33,34^ and an open port sampling interface coupled to ESI.^35^ In these levitation approaches, droplet generation is effectively decoupled from the chemical analysis step. Moreover, relatively precise control of environmental conditions (e.g., relative humidity, which controls solute concentration) as well as droplet size and charge are possible with these single droplet approaches. Free-flowing streams of picolitre droplets have also been coupled to ESI-MS to conduct low volume, high throughput chemical analysis.^36^ An important drawback to these approaches is that they require separate ionization stages, and, for many of them, the ionization process extends over several seconds, potentially reducing the sensitivity of the approach by spreading signal out over time and introducing artifacts (e.g., undesirable reactions) that may complicate measurement interpretation.^37^

Inlet ionization approaches have emerged over the past decade as straightforward and versatile methods to generate molecular ions from solid and liquid samples within the MS inlet, requiring minimal sample preparation and no use of high voltages or lasers.^38−44^ The samples can be introduced at ambient pressure, and the ions generated are similar to those observed using ESI. A recent advance on these inlet ionization approaches is droplet assisted ionization (DAI), where liquid aerosol droplets are sampled from ambient pressure into a heated capillary inlet attached to the MS.^45,46^ DAI has been used to measure the chemical composition of secondary organic aerosol and explore chemical reactivity in nanoparticles.^47,48^ While some uncertainty remains about the ionization mechanism, a liquid droplet is thought to undergo either thermal or aerodynamic breakup within the MS inlet, leading to charge separation and the formation of charged progeny droplets, followed by successive iterations of solvent evaporation and Coulombic fission to produce molecular ions.^45^ At low inlet temperatures, the droplet may flash freeze and shatter to generate molecular ions.^49^ This ionization approach holds promise for MS analysis of finite-volume liquid aerosol droplets but has not yet been applied to aerosol particles larger than ∼100 nm.

In this study, we describe a novel approach to focus, levitate, and sample individual picolitre volume droplets (∼2–180 pL, or ∼20–70 μm diameter) by coupling a linear quadrupole electrodynamic balance (LQ-EDB) with a high-resolution time-of-flight mass spectrometer using DAI. Droplet properties including size, charge, and droplet age are controlled. Strong MS signals for individual droplets containing as low as ∼1 pg analyte are detected. The factors contributing to the sensitivity of the approach are explored, and the suitability of the technique for a wide range of analytes from small molecules to biomolecules is discussed.

## Methods

**Figure 1:**
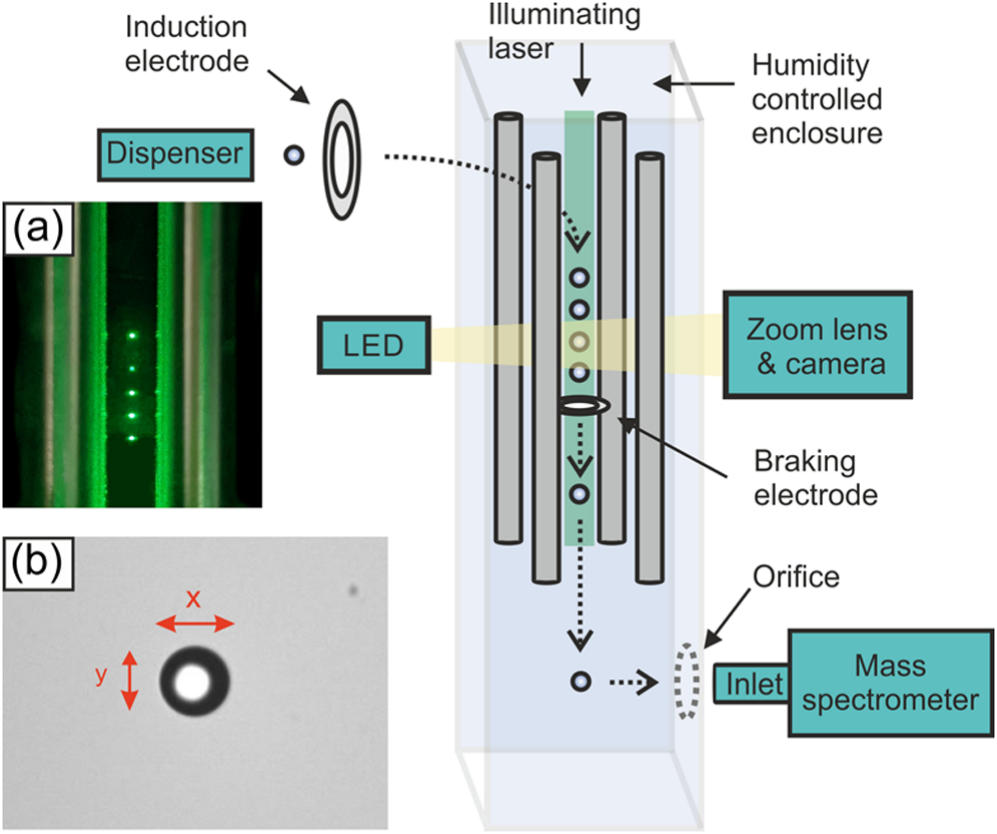
A schematic of the SDMS experimental setup. The inset images are (a) a photograph of five droplets stacked in the quadrupole and (b) an example brightfield image of a droplet trapped in the quadrupole.

A Single Droplet Mass Spectrometry (SDMS) approach to characterize the molecular composition of individual picolitre aerosol droplets was developed. Figure 1 illustrates the key aspects of the approach, whereas Figure S1 provides an image of the setup. Droplets of known composition and size are reproducibly generated using a droplet-on-demand (DoD) dispenser and guided to the MS inlet for subsequent chemical analysis using a linear quadrupole electrodynamic balance (LQ-EDB). Ionization is accomplished using DAI.^46^ An imaging assembly allows droplet size to be measured during transit through the LQ-EDB.

### Chemicals

Solutions for a range of small molecules and proteins were made for single droplet analysis. The systems examined include: angiotensin II (ApexBio), leucine enkephalin (Waters), cortisol, equine cytochrome-c, and equine myoglobin (Sigma-Aldrich). Solute concentrations and solvent compositions are described in the text.

### Droplet Generation

Individual picolitre droplets are generated with a DoD dispenser (MicroFab, MJ-ABP-01), a piezo-electric device that controllably and reproducibly generates droplets of a desired volume upon application of a voltage pulse. The range of droplet volumes accessible spans ∼2 to 180 pL (∼20–70 μm in diameter) and is determined by the orifice diameter of the dispenser and the voltage pulse applied to the dispenser. An induction electrode, positioned near the outlet orifice of the dispenser, imparts a small amount of charge (order of 10s of fC, corresponding to <5% of the Raleigh limit) to the droplets upon generation. The frequency of droplet generation is user-controlled and in this work was set to 1 Hz, except for the protein samples where 100 Hz was used. The 1 Hz frequency enabled multiple droplets to be measured over a short time period while ensuring baseline resolution in the MS signal between individual droplets.

### Droplet Control and Guidance

The LQ-EDB is positioned external to the MS in a vertical geometry and is held at ambient pressure. It traps the dispensed droplets and guides their trajectory toward the MS inlet. This arrangement ensures nearly 100% of the sample is delivered to the MS, minimizing sample wastage. The alternating electric field applied to the rods in the LQ-EDB confines the charged droplets in two lateral dimensions, collimating them as they transport along the central vertical axis.^50^ A charged droplet enters the quadrupole at the top of the central axis and descends along its full length with a velocity approaching the droplet’s terminal settling velocity. The LQ-EDB is housed within a transparent glass sheath, which contains openings for a gas inlet and outlet, droplet injection port, and droplet outlet to the MS inlet. The sheath inhibits air currents from disrupting droplet transport and permits control of environmental conditions (e.g., relative humidity, RH). In the experiments presented here, the RH was held as high as possible (>90% RH) by passing compressed air through a water bubbler before flowing the humidified gas through the enclosure. Because the ionization efficiency (discussed later) is sensitive to droplet water content, a high RH environment minimizes evaporation of the aqueous droplets during transit through the LQ-EDB. A braking electrode (applied with a voltage of the same polarity as the droplet charge) is positioned approximately halfway down the LQ-EDB. For the experiments described here, the braking electrode was activated immediately prior to performing a measurement, halting the droplet’s descent while its diameter was measured by the imaging setup. The braking electrode was then switched off to allow droplet sampling into the MS for chemical analysis.

Bespoke software, written in LabVIEW, controls all the electronic signals used in the LQ-EDB. The unamplified signals are generated using a multifunction DAQ (NI USB-6343) for the quadrupole rods, induction electrode, and braking electrode, and an arbitrary waveform generator (Keysight, Trueform 33500B Series) for the dispenser pulse. These signals are amplified using a dedicated amplification unit (Biral, P9050M), which allows real-time control of the quadrupole signal (between ±1 kVAC, 0–1 kHz), induction and braking electrode signals (between ±500 VDC), and dispenser pulse (∼40 V pulse height, ∼ 30 μs pulse width). In a typical experiment, the voltage applied to the quadrupole was ±1 kVAC with a 200 Hz frequency.

### Droplet Imaging

Droplets in the central axis of the LQ-EDB are illuminated with a low-power alignment laser (Thorlabs, 5 mW, 532 nm), allowing visual detection of the droplets, as shown in Figure 1a. In addition, a brightfield imaging assembly permits the droplets to be observed in more detail. This imaging assembly consists of an objective (Motic, Plan apo 20 × ), zoom and focusing module (Navitar, Resolv4K), and camera (JAI, GO-2400M-USB). Illumination is provided using an LED (Thorlabs, M455L3) with focusing lens. The image dimensions for each zoom setting were calibrated using a micrometre graticule. Collectively, the imaging system enables high resolution, high magnification visualization of individual droplets (Figure 1b). The droplet images permit relatively precise retrieval of droplet diameter (±2 μm) and, consequently, droplet volume.^51,52^

### Single Droplet Mass Spectrometry

The LQ-EDB assembly is coupled with a high-resolution quadrupole time-of-flight mass spectrometer (Waters, Synapt XS) by positioning the bottom opening of the LQ-EDB enclosure against the sample cone inlet of the mass spectrometer. After transiting the central axis of the LQ-EDB, droplets exit the electric field of the LQ-EDB and continue to fall vertically (∼10 mm) until they approach the MS inlet. The droplet is then aspirated into the MS inlet by the continuous ∼2 L/min MS inlet flow, where the droplet rapidly desolvates to generate molecular ions through an inlet ionization mechanism presumably similar to that proposed for DAI or solvent assisted ionization.^41,45^ For these experiments, the MS was operated in *sensitivity* mode to detect either positive or negative ions, with mass spectra typically collected over a 50–1200 *m*/*z* range (unless otherwise specified) with a 0.1 s scan time. Mass spectra were generated in centroid mode. The source conditions were optimized and held constant at the following values: source temperature 60–80 °C, sampling cone voltage 0 V, source offset voltage 30 V, trap and transfer collision energy off, capillary voltage (for ESI comparison experiments only) 1–1.5 kV.

## Results and Discussion

**Figure 2:**
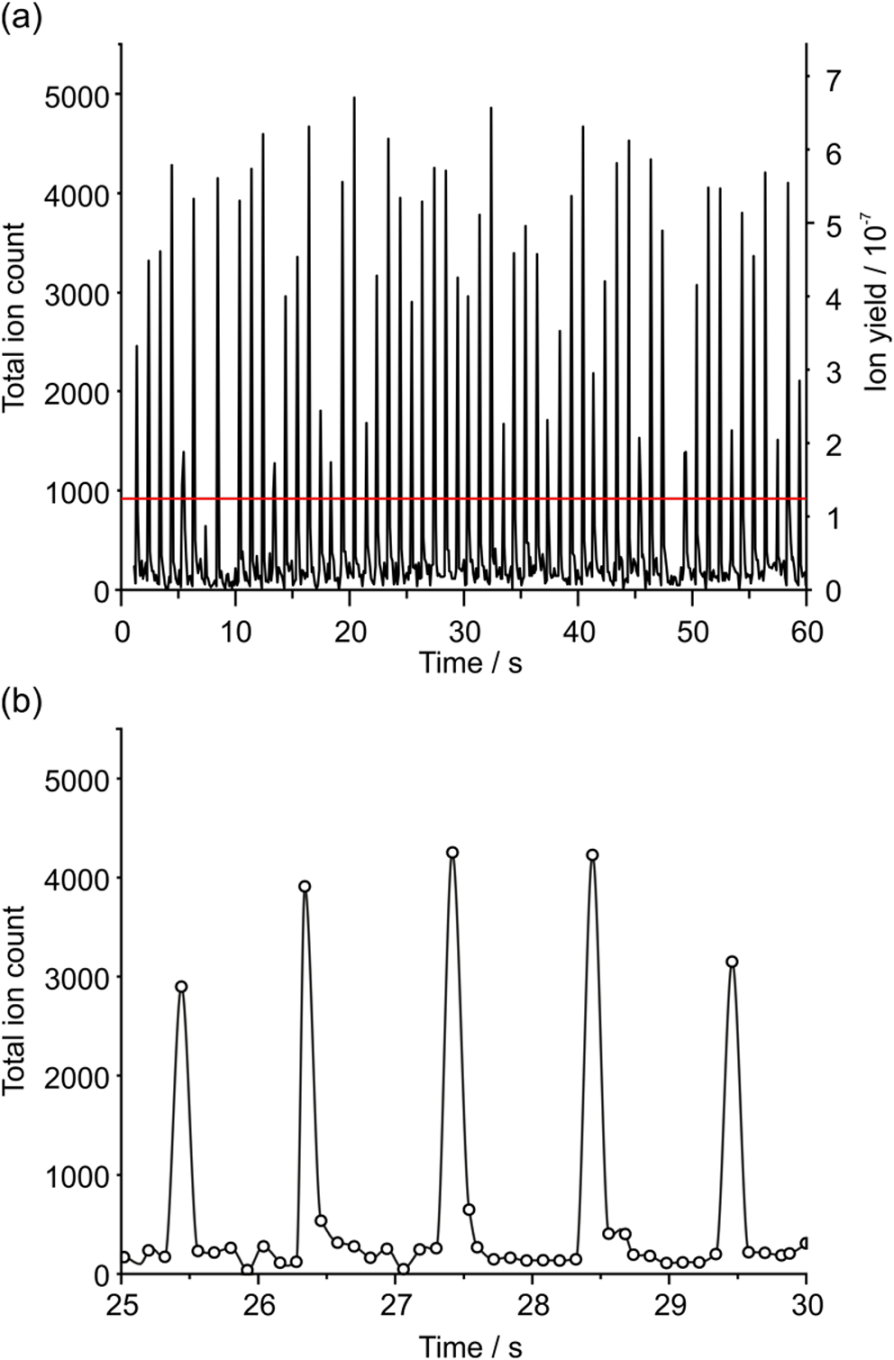
(a) Example chromatogram recorded for 39 pL droplets containing 320 μM angiotensin II, delivered to the MS at 1 Hz. The red line indicates a signal that is 20% of the largest measured signal. (b) A close-up of the same chromatogram, showing each droplet is analyzed within around one 0.1 s scan, with baseline resolution between sequential droplets.

An example total ion count (TIC) chromatogram recorded by the MS for a uniform series of 39 pL (42 μm diameter) aqueous droplets containing 320 μM angiotensin II (corresponding to 12.9 pg or 12 fmol of angiotensin II analyzed per droplet) dispensed at 1 Hz is shown in Figure 2a. The series of peaks at 1 s intervals in the chromatogram corresponds to analysis of individual picolitre droplets. Droplets generating very low TICs were observed to miss or clip the MS inlet due to small fluctuations in trajectory during the aspiration process. By defining a successful SDMS measurement as one that generates at least 20% of the largest measured signal across all droplets (red line in Figure 2a), the droplet hit rate approaches 95%, with a 33% standard deviation in maximum TIC intensity per droplet. The signal-to-noise ratios for the TICs of the detected droplets range between 4 and 16. Before delivery to the inlet, droplets have uniform size (<0.2%), charge (<3%), and composition.^53^ Therefore, observed variations in the TIC primarily arise from small variations in the efficiency of droplet sampling into the MS inlet or synchronization between droplet sampling and MS acquisition.^36^

Figure 2b magnifies a portion of this chromatogram, showing that virtually all ions arising from an individual droplet are contained within a single 0.1 s scan (the highest time resolution available on the MS using standard user settings), with baseline resolution achieved within 1–2 scans. This fast time scale for single droplet analysis is similar to that reported for a rapid droplet sampling interface where larger picolitre and nanolitre droplets are ejected onto an open-face capillary with a continuous flow of electrosprayed solvent.^36^ By contrast, single droplet analysis by other approaches (e.g., paper spray ionization or open port sampling interface), which rely on ejected droplets dissolving into larger volumes of solvent before electrospray, results in signal from a single droplet being spread out across at least an order of magnitude longer time scale (many seconds).^31,33,35,54^ The absence of analyte dilution within another solvent, along with the very short time scale over which analyte within an individual droplet is detected, suggests relatively high sensitivity for this approach compared to other single droplet analysis approaches.

**Figure 3:**
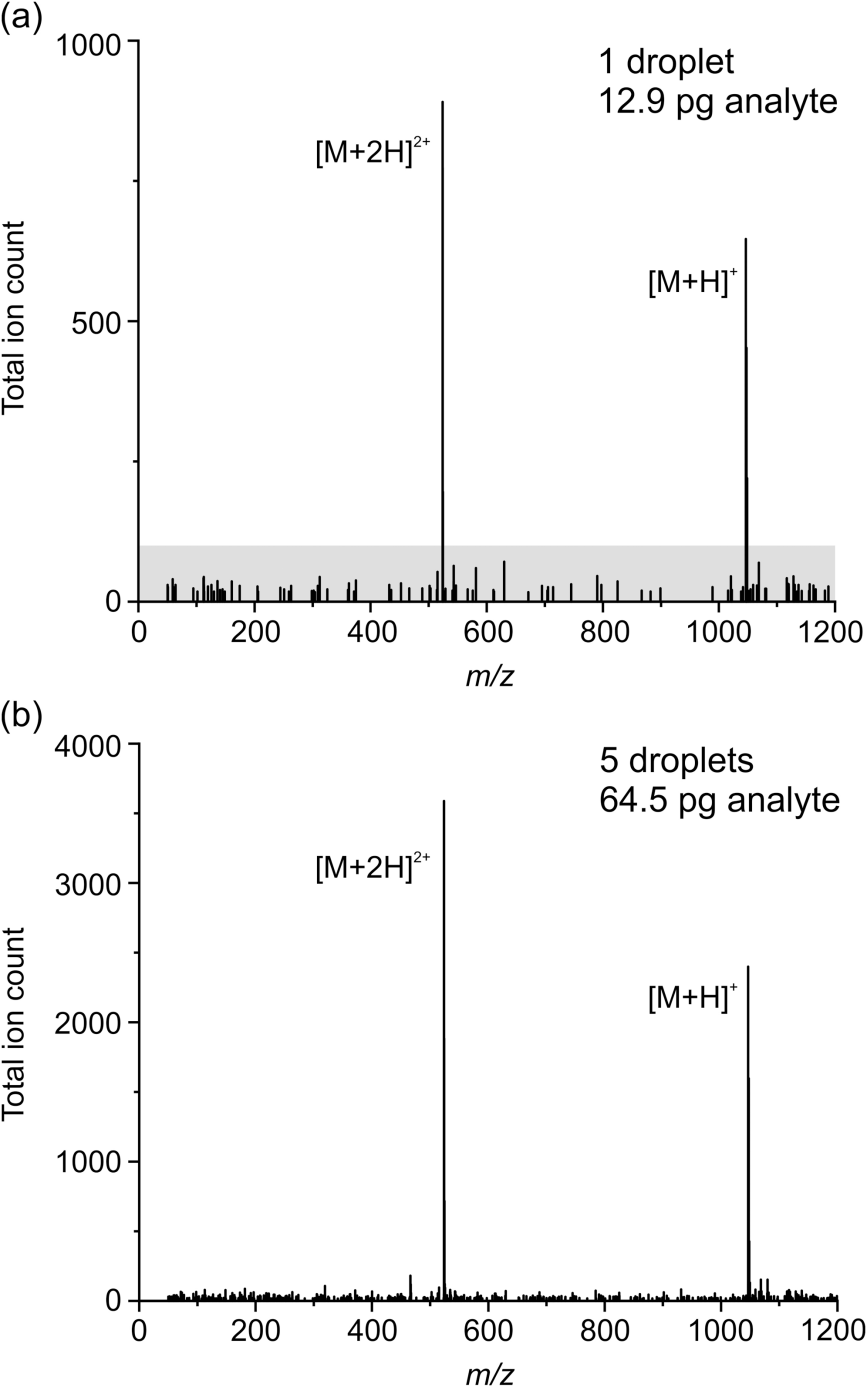
Example angiotensin II (320 μM) mass spectra (a) for a single 39 pL droplet and (b) integrated over 5 droplets. The gray shaded region in (a) corresponds to a TIC < 100.

Representative mass spectra for the droplets detected in Figure 2 are shown in Figure 3. Figure 3a shows a single droplet mass spectrum for angiotensin II, with [M + H]^+^ and [M+2H]^2+^ molecular ions at 1047 *m*/*z* and 524 *m/*z, respectively, clearly resolvable from the noise. The magnitude of the noise envelope (i.e., the magnitude of the largest nonmolecular ion peak) is <8% of the [M+2H]^2+^ signal. Owing to droplet-to-droplet variation in signal intensity (see Figure 2), some improvement in the cleanliness of the mass spectrum arises from averaging signal from multiple droplets. Figure 3b shows that by integrating signal across 5 droplets (64.5 pg of analyte consumed) the mass spectral noise is reduced to <5% of the [M+2H]^2+^ ion. Integrating over additional droplets leads to diminishing reductions in spectral noise: integrating across all 60 droplets in Figure 2, the noise level is still 4.4% of the [M+2H]^2+^ ion signal. Moreover, Figure S2c shows an example background (no droplet) mass spectrum taken between delivery of two droplets, highlighting the very low background signal observed between droplets. Clear mass spectra for individual droplets containing pg quantities of analyte are also detected for leucine-enkephalin and cortisol (Figure S2).

Analysis of all droplets detected in Figure 2 shows that the relative abundance of the +1 and +2 charge states of the molecular ion exhibit little variation between droplets (Figure S3), with the +2 charge state accounting for 65 ± 9% of the all molecular ions detected. Perhaps unsurprisingly, the largest droplet-to-droplet variations in the relative intensities occurs for droplet mass spectra where the total ion count is low. Additionally, isotopic distributions for single droplets can be clearly resolved (Figure S4). Although there is a slight deviation from expected isotopic abundances for a single droplet, after integrating across only 5 droplets the isotopic abundance is consistent with expected values.

### Comparisons to ESI

**Figure 4:**
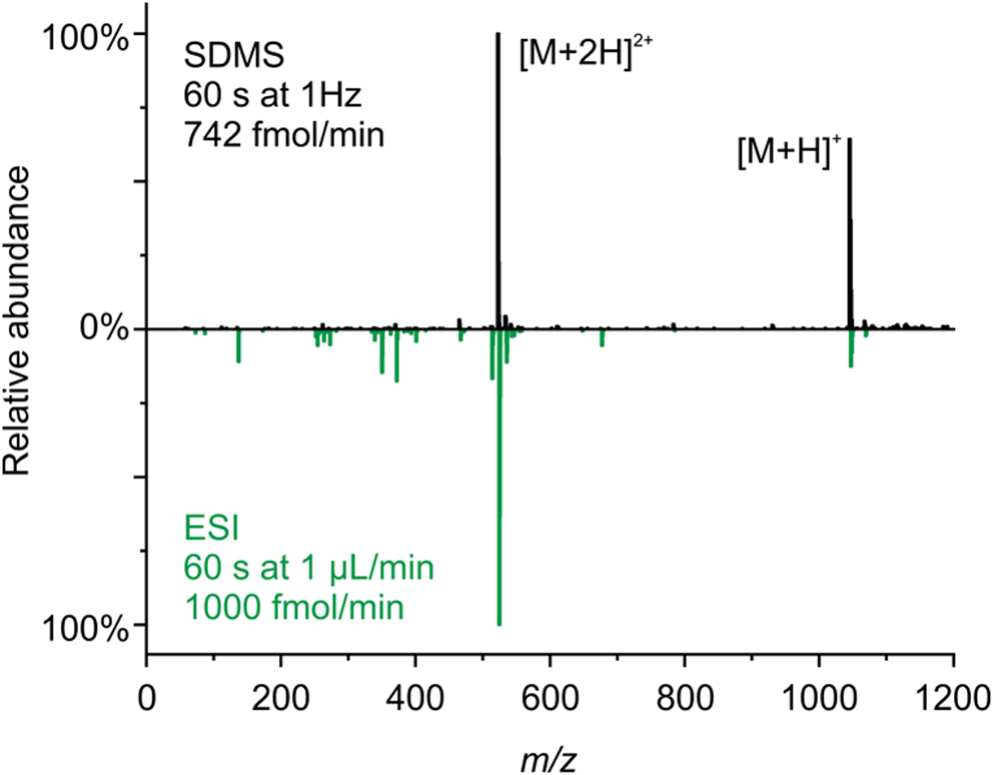
Comparison of angiotensin II signal integrated over 60 s using the SDMS approach and ESI.

Despite fundamental differences in the two approaches, it is instructive to compare SDMS experiments (where individual droplets are analyzed at discrete intervals) and ESI experiments (where analyte flow to the MS is continuous), as this allows evaluation of the relative benefits of each approach. Figure 4 shows SDMS and ESI mass spectra for angiotensin II collected under similar MS conditions. Both mass spectra are integrated over a 60 s measurement window, with the SDMS apparatus generating droplets at 1 Hz using 320 μM aqueous solution and with ESI sampling at a flow rate of 1 μL/min using a more dilute 1 μM aqueous solution. These two setups enable comparison at similar analyte molar flow rates (742 fmol/min for SDMS, and 1000 fmol/min for ESI). The SDMS mass spectra are clean and exhibit relatively more ion signal from [M + H]^+^ compared to ESI, where most of the molecular ion signal is detected as [M + H]^2+^. In this specific comparison, the ion yields (number of analyte ions detected divided by the number of analyte molecules delivered to the MS) for SDMS and ESI are 2.3 × 10^–7^ and 6 × 10^–5^, respectively, indicating that SDMS at these specific conditions is 260 times less sensitive on a per-molecule basis than ESI. At these conditions, the SDMS approach involves a significantly larger droplet size, higher analyte concentration, and much lower level of droplet charge compared to ESI. Despite the lower ion yield for SDMS, the low absolute amount of sample consumed and the ability to time delivery of the droplet to the MS provides significant benefits. Moreover, as discussed in the next section, the ion yield for the SDMS approach can be improved substantially by altering droplet size and analyte concentration.

### Size, Concentration, and Charge Dependencies of Ion Yield

**Figure 5:**
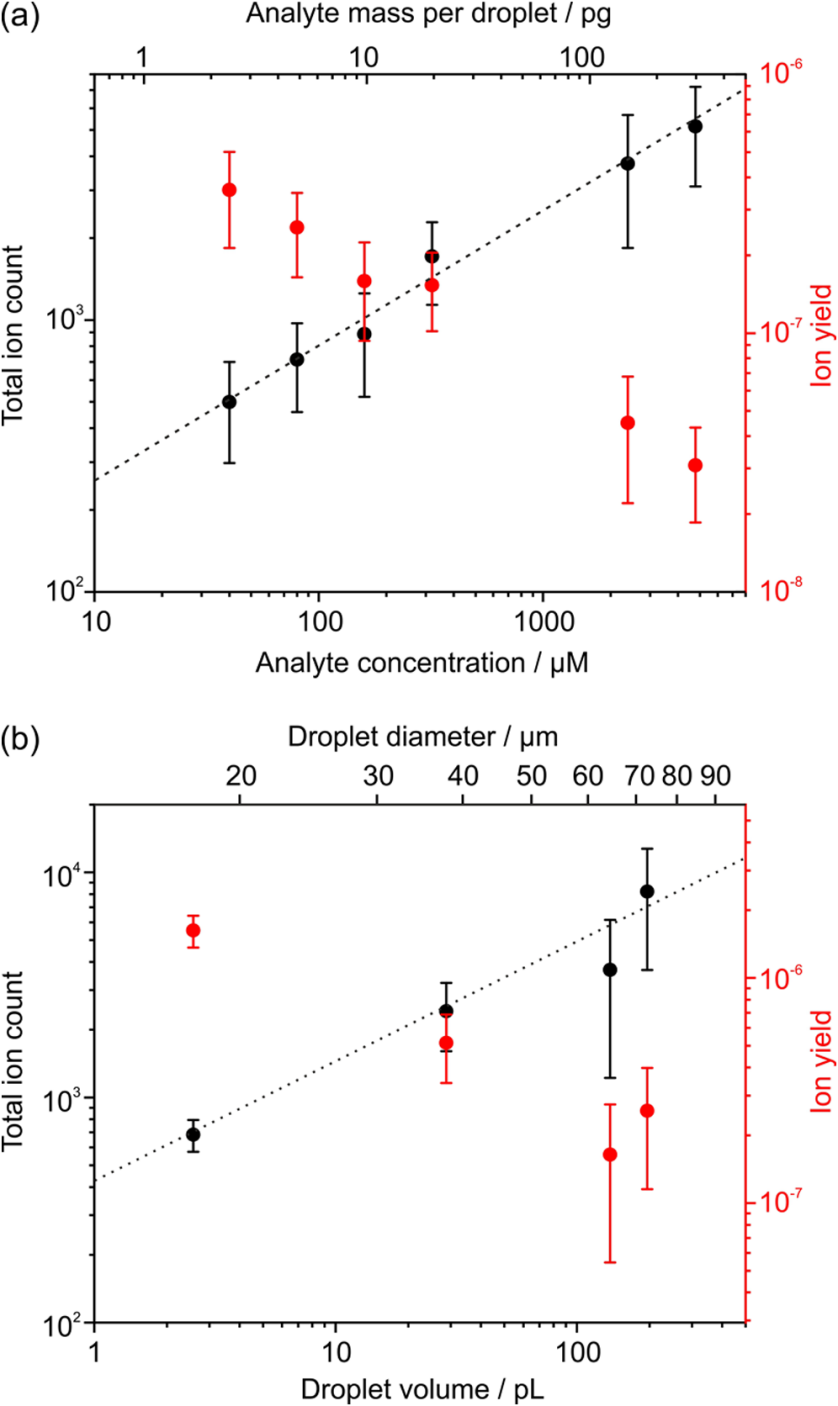
Measured ion count and ion yield as a function of (a) analyte concentration (droplet volume held constant at 58 pL) and (b) droplet volume (analyte concentration held constant at 320 μM). The black dashed lines indicate a log–log relationship between ion count and (a) analyte concentration and (b) droplet volume.

We next show how the SDMS ion yield varies with analyte concentration and droplet volume. These experiments also provide key information about the ionization mechanism. Figure 5 shows the measured TIC and ion yield as a function of angiotensin II concentration (holding droplet volume constant at 58 pL, Figure 5a) and as a function of droplet volume (holding analyte concentration constant at 320 μM, Figure 5b). Each data point represents the average value calculated across all detected droplets in a 60 s measurement window. Error bars represent the standard deviation.

Across nearly 3 orders of magnitude in analyte concentration, increasing analyte concentration within a droplet increases the TIC. At the same time, the ion yield per droplet decreases with increasing analyte concentration. This observation highlights the importance of the protic solvent water to the ionization process. For instance, when analyzing plumes of submicron aerosol, drying the aerosol to low relative humidity or using a nonpolar solvent resulted in orders-of-magnitude decreases in ion signal.^45,46^ The dependence of ion yield on analyte concentration reflects the ability of water to donate charge to the analyte as well as its apparent ability to induce charge separation during droplet breakup.^55^ Consequently, although a higher absolute analyte mass in a droplet leads to a greater signal intensity, the ionization process becomes less efficient, suggesting the SDMS approach is best suited to analysis of dilute droplets. In the current configuration of the approach, aqueous droplets containing down to 2.4 pg analyte in a 58 pL volume (48 μm diameter) droplet were successfully analyzed with good analyte intensity. Droplets containing lower masses of analyte were not routinely observed, mainly due to challenges in optimizing droplet delivery, as such droplets individually generate relatively little signal (owing to the small amount of analyte they contain). However, there are multiple avenues to improve the absolute signal (e.g., by sampling droplets through a heated capillary or by improving alignment of the LQ-EDB with the MS inlet), which will be explored in future work. Extrapolating of the log–log relationship shown in Figure 5a and assuming the absolute level of background noise remains constant across all droplet sizes (at ∼100 counts), the current measured limit-of-detection could hypothetically be improved to ∼100 fg analyte. We note that if this approach were utilized to explore chemical kinetics in aerosol droplets, reactions would generally occur at a fixed RH, mitigating any RH-dependence on the sensitivity of the approach.^46^

Similarly, droplet volume also plays a key role in the ionization process. As droplet volume increases from 2.5 pL to 195 pL, the TIC increases by approximately 1 order of magnitude. This increase is because the total analyte mass delivered to the MS increases from 0.9 pg to 65.1 pg across the investigated droplet volume range. By contrast, ion yields are about an order of magnitude larger for the 2.5 pL volume droplets compared to the 195 pL droplets. In other words, ionization is more efficient for smaller droplets. This observation is consistent with size-dependent analyses previously performed on submicron particles analyzed using DAI, demonstrating that analytes in smaller droplets are more efficiently ionized,^46,47^ as well as with trends in ionization efficiency using (nano-)ESI.^56,57^ In the current configuration, the minimum droplet size is limited by what can be generated using the DoD dispenser. However, extrapolating the observed trends suggests that single droplet analysis is in principle feasible at least until the droplet volume falls below 100 fL (i.e., 5 μm diameter) for angiotensin II droplets at the same concentration, an extrapolation roughly consistent with analysis of ionization trends for plumes of submicron aerosols.^45^ Note that when single droplet analysis becomes challenging, signal can still be detected by integrating across multiple droplets.

The log–log plots of the TIC dependence on analyte concentration and droplet volume in Figure 5 are both linear with a gradient 0.5. The ion yields presented in Figure 5 fall between 10^–6^ and 10^–8^ across the studied ranges in droplet volume and angiotensin II concentration. Given the observed increase in ion yield with both decreasing volume and decreasing analyte concentration, ion yields better than 10^–5^ could potentially be achieved in the future by leveraging these phenomena in concert. These ion yields compare very favorably to those calculated for submicron plumes under similar conditions, ∼ 10^–9^,^45^ and even approach the ion yields achieved using ESI under similar conditions, ∼ 10^–5^, despite the far lower absolute quantities of analyte required in SDMS and absence of highly charged droplets. Indeed, since individual picolitre droplets can be detected using this approach, only a tiny fraction of the bulk volume used to load the dispenser is consumed in a typical experiment and any unused sample can be extracted for other purposes.

We also explored the relative importance of droplet charge on ionization using the SDMS approach. As described earlier, a small amount of charge is imparted onto the droplets to collimate them using the LQ-EDB. The total charge is limited by the maximum induction electrode voltage, which imparts 10s of fC per droplet (i.e., < 5% of the Rayleigh limit for 50 pL droplets). Droplet charge was systematically varied between ±100 fC and quantified by depositing droplets into a Faraday cup that was positioned below the LQ-EDB and connected to an electrometer (Keithly 6514/E). For angiotensin II, both positive and negative molecular ions are detectable, irrespective of the polarity of the charge initially applied to the droplet, although in general more positive ions were detected than negative ions (Figure S5). Total ion count does not vary significantly with net initial droplet charge, consistent with the hypothesis that the protic solvent and droplet breakup process (rather than initial droplet charge) are key to ionization. It is possible that droplet charge may play a minor role in the ionization process but its effects are obscured by droplet-to-droplet variations in ion signal over the investigated range of droplet net charge.

### Protein Analysis

**Figure 6:**
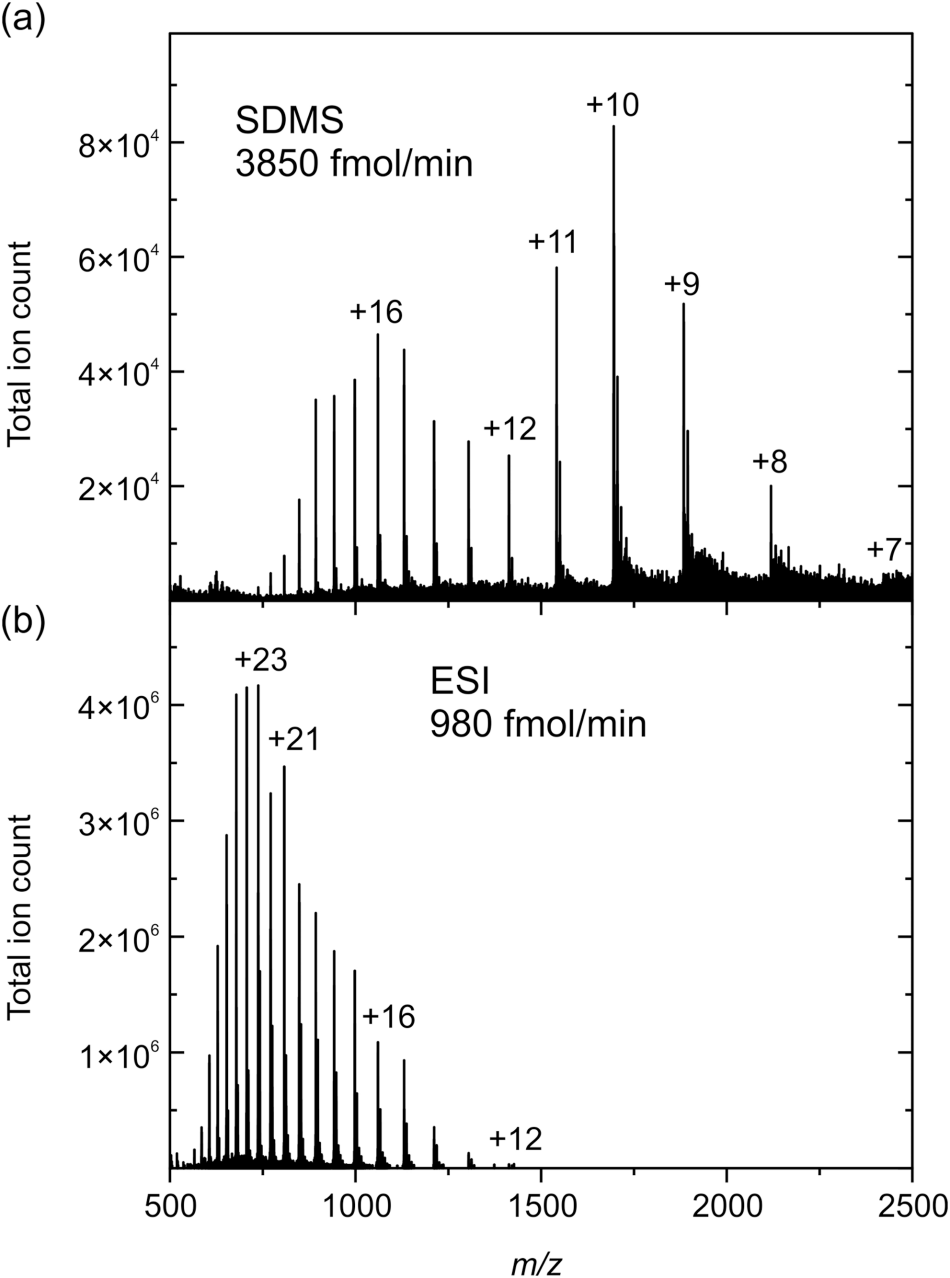
Example 60 s myoglobin mass spectra for (a) SDMS (50 μm droplet diameter, 10 μM + 0.1% formic acid) and (b) ESI (1 μM + 0.1% formic acid).

Finally, we explore the capability of SDMS for detecting proteins (>10 kDa). In these experiments, the droplet dispensing frequency was increased to 100 Hz to allow comparison with ESI at similar volumetric flow rates (1 μL/min ESI, ∼ 0.4 μL/min SDMS). Figure 6 shows example 60 s myoglobin mass spectra for SDMS (3850 fmol/min flow rate) and ESI (980 fmol/min flow rate). The SDMS mass spectrum exhibits two clear charge state envelopes, presumably corresponding to unfolded (higher charge states) and folded (lower charge states) proteins,^58^ with the low charge state tail extending to high *m*/*z* ions where no resolvable analyte signal is detected by ESI. For SDMS, the weighted average charge state is 13.6, whereas for ESI the weighted average charge state is 22.1. This preference for lower charge states in SDMS compared to ESI is also clearly observed for cytochrome-C (Figure S6) and presumably arises because the analysis is charge-limited (i.e., a limited amount of charge exists to partition across protein molecules in the droplet). In Figure 6, the total ion yield across all observed charge states (∼1 × 10^–6^ for SDMS and ∼2 × 10^–4^ for ESI) suggests that the sensitivity of the current SDMS configuration is around 100 times lower than that of ESI, consistent with the observations reported in Figure 3 for small molecules. However, as discussed above, judicious choice of droplet volume and analyte concentration may bring the SDMS ion yields closer to parity with ESI. Moreover, the weighted average charge state and ion yield values using SDMS for these proteins are much closer to those measured in ammonium acetate buffer using ESI (Figure S7).

## Conclusions

A novel approach for MS of individual picolitre volume droplets is reported. Individual droplets are generated by a droplet dispenser, imparted a small amount of net charge, and guided to the MS inlet using a LQ-EDB. Analyte molecules are ionized using DAI, where the polar solvent in a liquid droplet imparts charge to the analyte molecules contained within during droplet breakup within the MS inlet. There are several benefits of this approach to analyze picolitre droplets. Sample preparation is minimal, and the droplet generation and manipulation processes are segregated from the ionization and analysis steps. Indeed, the chemical analysis step is complete in milliseconds within the MS inlet, compared to chemical analysis requiring several seconds to tens of seconds for comparable single droplet MS approaches, reducing the potential for artifacts arising from the ionization process (e.g., further reaction) affecting measurement interpretation. The approach can characterize droplets across a wide volume range (∼2–180 pL) currently limited only by the droplet generation technique. This SDMS approach permits efficient chemical analysis of individual droplets containing low pg quantities of analyte. Although the approach in its current configuration is somewhat less sensitive than ESI in terms of ion yield (10^–7^–10^–5^ for SDMS vs 10^–5^ for ESI), the SDMS approach generates clean mass spectra and permits timing of droplet delivery to the MS, potentially yielding benefits over continuous flow approaches for applications using mass spectrometers with a lower duty cycle. The ability to detect proteins was demonstrated, with the detected protein ions exhibiting lower charge states than in comparable ESI mass spectra.

This SDMS approach will facilitate explorations of the factors governing chemical reactions in aerosol droplets. Such studies will improve our mechanistic understanding of atmospheric aerosol and cloud droplet chemistry. Additionally, this SDMS technique may leverage the reaction accelerations inherent in aerosol droplets for reaction discovery and optimization processes. The capability to generate low-charge state protein ions may also facilitate new insights into protein structures. Lastly, the approach may also have broader utility for efficient analysis of picoscopic samples in different application domains.

## Data Availability

All data underlying the figures are available through the University of Bristol data repository, data.bris, at https://doi.org/10.5523/bris.1zjrkhdd6cstr2hj6yn4it9jze.

## Supporting Information Available

Image of the SDMS setup, individual picodroplet mass spectra for leucine-enkephalin and cortisol, charge state abundances for angiotensin II, isotope distribution for angiotensin II, droplet charge effects on ion count, SDMS mass spectra of cytochrome-C, ESI mass spectra of myoglobin and cytochrome-C with ammonium acetate buffer (PDF)

## References

[ref1] Bzdek B. R.; Reid J. P.; Cotterell M. I. Open Questions on the Physical Properties of Aerosols. Commun. Chem. 2020, 3, 105 10.1038/s42004-020-00342-9. 36703389 PMC9814152

[ref2] Arroyo P. C.; David G.; Alpert P. A.; Parmentier E. A.; Ammann M.; Signorell R. Amplification of Light within Aerosol Particles Accelerates In-Particle Photochemistry. Science 2022, 376, 293–296. 10.1126/science.abm7915. 35420964

[ref3] Bain R. M.; Pulliam C. J.; Thery F.; Cooks R. G. Accelerated Chemical Reactions and Organic Synthesis in Leidenfrost Droplets. Angew. Chem., Int. Ed. 2016, 55, 10478–10482. 10.1002/anie.201605899. 27465311

[ref4] Zhong X.; Chen H.; Zare R. N. Ultrafast Enzymatic Digestion of Proteins by Microdroplet Mass Spectrometry. Nat. Commun. 2020, 11, 1049 10.1038/s41467-020-14877-x. 32103000 PMC7044307

[ref5] Yan X. Emerging Microdroplet Chemistry for Synthesis and Analysis. Int. J. Mass Spectrom. 2021, 468, 116639 10.1016/j.ijms.2021.116639.

[ref6] Wilson K. R.; Prophet A. M.; Rovelli G.; Willis M. D.; Rapf R. J.; Jacobs M. I. A Kinetic Description of How Interfaces Accelerate Reactions in Micro-Compartments. Chem. Sci. 2020, 11, 8533–8545. 10.1039/D0SC03189E. 34123113 PMC8163377

[ref7] Fallah-Araghi A.; Meguellati K.; Baret J.-C.; Harrak A. E.; Mangeat T.; Karplus M.; Ladame S.; Marques C. M.; Griffiths A. D. Enhanced Chemical Synthesis at Soft Interfaces: A Universal Reaction-Adsorption Mechanism in Microcompartments. Phys. Rev. Lett. 2014, 112, 028301 10.1103/PhysRevLett.112.028301. 24484045

[ref8] Narendra N.; Chen X.; Wang J.; Charles J.; Cooks R. G.; Kubis T. Quantum Mechanical Modeling of Reaction Rate Acceleration in Microdroplets. J. Phys. Chem. A 2020, 124, 4984–4989. 10.1021/acs.jpca.0c03225. 32453564

[ref9] Xiong H.; Lee J. K.; Zare R. N.; Min W. Strong Concentration Enhancement of Molecules at the Interface of Aqueous Microdroplets. J. Phys. Chem. B 2020, 124, 9938–9944. 10.1021/acs.jpcb.0c07718. 33084345

[ref10] Rovelli G.; Jacobs M. I.; Willis M. D.; Rapf R. J.; Prophet A. M.; Wilson K. R. A Critical Analysis of Electrospray Techniques for the Determination of Accelerated Rates and Mechanisms of Chemical Reactions in Droplets. Chem. Sci. 2020, 11, 13026–13043. 10.1039/D0SC04611F. 34094487 PMC8163298

[ref11] Chen C. J.; Williams E. R. The Role of Analyte Concentration in Accelerated Reaction Rates in Evaporating Droplets. Chem. Sci. 2023, 14, 4704–4713. 10.1039/D3SC00259D. 37181782 PMC10171075

[ref12] Heindel J. P.; LaCour R. A.; Head-Gordon T. The Role of Charge in Microdroplet Redox Chemistry. Nat. Commun. 2024, 15, 3670 10.1038/s41467-024-47879-0. 38693110 PMC11519639

[ref13] Banerjee S.; Zare R. N. Syntheses of Isoquinoline and Substituted Quinolines in Charged Microdroplets. Angew. Chem., Int. Ed. 2015, 54, 14795–14799. 10.1002/anie.201507805. 26450661

[ref14] Lee J. K.; Kim S.; Nam H. G.; Zare R. N. Microdroplet Fusion Mass Spectrometry for Fast Reaction Kinetics. Proc. Natl. Acad. Sci. U. S. A. 2015, 112, 3898–3903. 10.1073/pnas.1503689112. 25775573 PMC4386409

[ref15] Laskin J.; Eckert P. A.; Roach P. J.; Heath B. S.; Nizkorodov S. A.; Laskin A. Chemical Analysis of Complex Organic Mixtures Using Reactive Nanospray Desorption Electrospray Ionization Mass Spectrometry. Anal. Chem. 2012, 84, 7179–7187. 10.1021/ac301533z. 22812571

[ref16] Girod M.; Moyano E.; Campbell D. I.; Cooks R. G. Accelerated Bimolecular Reactions in Microdroplets Studied by Desorption Electrospray Ionization Mass Spectrometry. Chem. Sci. 2011, 2, 501–510. 10.1039/C0SC00416B.

[ref17] Iyer K.; Yi J.; Bogdan A.; Talaty N.; Djuric S. W.; Cooks R. G. Accelerated Multi-Reagent Copper Catalysed Coupling Reactions in Micro Droplets and Thin Films. React. Chem. Eng. 2018, 3, 206–209. 10.1039/C8RE00002F.

[ref18] Pratt K. A.; Prather K. A. Mass Spectrometry of Atmospheric Aerosols—Recent Developments and Applications. Part II: On-Line Mass Spectrometry Techniques. Mass Spectrom. Rev. 2012, 31, 17–48. 10.1002/mas.20330. 21449003

[ref19] Bzdek B. R.; Pennington M. R.; Johnston M. V. Single Particle Chemical Analysis of Ambient Ultrafine Aerosol: A Review. J. Aerosol Sci. 2012, 52, 109–120. 10.1016/j.jaerosci.2012.05.001.

[ref20] Hinz K. P.; Spengler B. Instrumentation, Data Evaluation and Quantification in on-Line Aerosol Mass Spectrometry. J. Mass Spectrom. 2007, 42, 843–860. 10.1002/jms.1262. 17589890

[ref21] Johnston M. V. Sampling and Analysis of Individual Particles by Aerosol Mass Spectrometry. J. Mass Spectrom. 2000, 35, 585–595. 10.1002/(SICI)1096-9888(200005)35:5<585::AID-JMS992>3.0.CO;2-K. 10800047

[ref22] Warschat C.; Stindt A.; Panne U.; Riedel J. Mass Spectrometry of Levitated Droplets by Thermally Unconfined Infrared-Laser Desorption. Anal. Chem. 2015, 87, 8323–8327. 10.1021/acs.analchem.5b01495. 26165504

[ref23] Warschat C.; Riedel J. Studying the Field Induced Breakup of Acoustically Levitated Drops. Rev. Sci. Instrum. 2017, 88, 105108 10.1063/1.5004046. 29092498

[ref24] Mu C.; Wang J.; Barraza K. M.; Zhang X.; Beauchamp J. L. Mass Spectrometric Study of Acoustically Levitated Droplets Illuminates Molecular-Level Mechanism of Photodynamic Therapy for Cancer Involving Lipid Oxidation. Angew. Chem., Int. Ed. 2019, 58, 8082–8086. 10.1002/anie.201902815. 31016864

[ref25] Grimm R. L.; Hodyss R.; Beauchamp J. L. Probing Interfacial Chemistry of Single Droplets with Field-Induced Droplet Ionization Mass Spectrometry: Physical Adsorption of Polycyclic Aromatic Hydrocarbons and Ozonolysis of Oleic Acid and Related Compounds. Anal. Chem. 2006, 78, 3800–3806. 10.1021/ac0601922. 16737240

[ref26] Westphall M. S.; Jorabchi K.; Smith L. M. Mass Spectrometry of Acoustically Levitated Droplets. Anal. Chem. 2008, 80, 5847–5853. 10.1021/ac800317f. 18582090 PMC2561267

[ref27] Van Wasen S.; You Y.; Beck S.; Riedel J.; Volmer D. A. Quantitative Analysis of Pharmaceutical Drugs Using a Combination of Acoustic Levitation and High Resolution Mass Spectrometry. Anal. Chem. 2021, 93, 6019–6024. 10.1021/acs.analchem.1c00762. 33835771

[ref28] Van Wasen S.; You Y.; Beck S.; Riedel J.; Volmer D. A. Laser Ablation Secondary Electrospray Ionization for in Situ Mass Spectrometric Interrogation of Acoustically-Levitated Droplets. Anal. Chem. 2022, 94, 16992–16996. 10.1021/acs.analchem.2c03800. 36450044

[ref29] Crawford E. A.; Esen C.; Volmer D. A. Real Time Monitoring of Containerless Microreactions in Acoustically Levitated Droplets via Ambient Ionization Mass Spectrometry. Anal. Chem. 2016, 88, 8396–8403. 10.1021/acs.analchem.6b01519. 27505037

[ref30] Kohli R. K.; Davies J. F. Paper Spray Mass Spectrometry for the Analysis of Picoliter Droplets. Analyst 2020, 145, 2639–2648. 10.1039/C9AN02534K. 32064475

[ref31] Jacobs M. I.; Davies J. F.; Lee L.; Davis R. D.; Houle F.; Wilson K. R. Exploring Chemistry in Microcompartments Using Guided Droplet Collisions in a Branched Quadrupole Trap Coupled to a Single Droplet, Paper Spray Mass Spectrometer. Anal. Chem. 2017, 89, 12511–12519. 10.1021/acs.analchem.7b03704. 29048875

[ref32] Birdsall A. W.; Krieger U. K.; Keutsch F. N. Electrodynamic Balance-Mass Spectrometry of Single Particles as a New Platform for Atmospheric Chemistry Research. Atmos. Meas. Technol. 2018, 11, 33–47. 10.5194/amt-11-33-2018.

[ref33] Willis M. D.; Rovelli G.; Wilson K. R. Combining Mass Spectrometry of Picoliter Samples with a Multicompartment Electrodynamic Trap for Probing the Chemistry of Droplet Arrays. Anal. Chem. 2020, 92, 11943–11952. 10.1021/acs.analchem.0c02343. 32786501

[ref34] Müller M.; Mishra A.; Berkemeier T.; Hausammann E.; Peter T.; Krieger U. K. Electrodynamic Balance–Mass Spectrometry Reveals Impact of Oxidant Concentration on Product Composition in the Ozonolysis of Oleic Acid. Phys. Chem. Chem. Phys. 2022, 24, 27086–27104. 10.1039/D2CP03289A. 36326041

[ref35] Kaur Kohli R.; Van Berkel G. J.; Davies J. F. An Open Port Sampling Interface for the Chemical Characterization of Levitated Microparticles. Anal. Chem. 2022, 94, 3441–3445. 10.1021/acs.analchem.1c05550. 35167275

[ref36] Cahill J. F.; Kertesz V. Rapid Droplet Sampling Interface for Low-Volume, High-Throughput Mass Spectrometry Analysis. Anal. Chem. 2023, 95, 16418–16425. 10.1021/acs.analchem.3c04015. 37888790

[ref37] Prophet A. M.; Polley K.; Van Berkel G. J.; Limmer D. T.; Wilson K. R. Iodide Oxidation by Ozone at the Surface of Aqueous Microdroplets. Chem. Sci. 2024, 15, 736–756. 10.1039/D3SC04254E. 38179528 PMC10762724

[ref38] Trimpin S.; Inutan E. D.; Pagnotti V. S.; Karki S.; Marshall D. D.; Hoang K.; Wang B.; Lietz C. B.; Richards A. L.; Yenchick F. S.; Lee C.; et al. Direct Sub-Atmospheric Pressure Ionization Mass Spectrometry: Evaporation/Sublimation-Driven Ionization Is Amazing, Fundamentally, and Practically. J. Mass Spectrom. 2024, 59, e5018 10.1002/jms.5018. 38736378 PMC12105868

[ref39] Trimpin S. Magicundefined Ionization Mass Spectrometry. J. Am. Soc. Mass Spectrom. 2016, 27, 4–21. 10.1007/s13361-015-1253-4. 26486514 PMC4686549

[ref40] McEwen C. N.; Pagnotti V. S.; Inutan E. D.; Trimpin S. New Paradigm in Ionization: Multiply Charged Ion Formation from a Solid Matrix without a Laser or Voltage. Anal. Chem. 2010, 82, 9164–9168. 10.1021/ac102339y. 20973512

[ref41] Pagnotti V. S.; Inutan E. D.; Marshall D. D.; McEwen C. N.; Trimpin S. Inlet Ionization: A New Highly Sensitive Approach for Liquid Chromatography/Mass Spectrometry of Small and Large Molecules. Anal. Chem. 2011, 83, 7591–7594. 10.1021/ac201982r. 21899326

[ref42] Hirabayashi A.; Sakairi M.; Koizumi H. Sonic Spray Mass Spectrometry. Anal. Chem. 1995, 67, 2878–2882. 10.1021/ac00113a023. 8779414

[ref43] Pei J.; Yu K.; Wang Y. Thermal Bursting Ionization for Ambient Mass Spectrometry. RSC Adv. 2016, 6, 2496–2499. 10.1039/C5RA22626K.

[ref44] Wleklinski M.; Li Y.; Bag S.; Sarkar D.; Narayanan R.; Pradeep T.; Cooks R. G. Zero Volt Paper Spray Ionization and Its Mechanism. Anal. Chem. 2015, 87, 6786–6793. 10.1021/acs.analchem.5b01225. 26024306

[ref45] Apsokardu M. J.; Kerecman D. E.; Johnston M. V. Ion Formation in Droplet-Assisted Ionization. Rapid Commun. Mass Spectrom. 2021, 35 (S1), e8227 10.1002/rcm.8227. 29971846

[ref46] Horan A. J.; Apsokardu M. J.; Johnston M. V. Droplet Assisted Inlet Ionization for Online Analysis of Airborne Nanoparticles. Anal. Chem. 2017, 89, 1059–1062. 10.1021/acs.analchem.6b04718. 28194981

[ref47] Kerecman D. E.; Apsokardu M. J.; Talledo S. L.; Taylor M. S.; Haugh D. N.; Zhang Y.; Johnston M. V. Online Characterization of Organic Aerosol by Condensational Growth into Aqueous Droplets Coupled with Droplet-Assisted Ionization. Anal. Chem. 2021, 93, 2793–2801. 10.1021/acs.analchem.0c03697. 33513002

[ref48] Zhang Y.; Apsokardu M. J.; Kerecman D. E.; Achtenhagen M.; Johnston M. V. Reaction Kinetics of Organic Aerosol Studied by Droplet Assisted Ionization: Enhanced Reactivity in Droplets Relative to Bulk Solution. J. Am. Soc. Mass Spectrom. 2021, 32, 46–54. 10.1021/jasms.0c00057. 32469218

[ref49] Apsokardu M. J.; Krasnomowitz J. M.; Jiang S.; Johnston M. V. Ion Formation from Rapidly Heated Aqueous Droplets by Droplet-Assisted Ionization. J. Phys. Chem. A 2020, 124, 7313–7321. 10.1021/acs.jpca.0c07101. 32833452

[ref50] Davies J. F. Mass, Charge, and Radius of Droplets in a Linear Quadrupole Electrodynamic Balance. Aerosol Sci. Technol. 2019, 53, 309–320. 10.1080/02786826.2018.1559921.

[ref51] Hardy D. A.; Archer J.; Lemaitre P.; Vehring R.; Reid J. P.; Walker J. S. High Time Resolution Measurements of Droplet Evaporation Kinetics and Particle Crystallisation. Phys. Chem. Chem. Phys. 2021, 23, 18568–18579. 10.1039/D1CP02840E. 34612393

[ref52] McCarthy L. P.; Reid J. P.; Walker J. S. High Frame-Rate Imaging of the Shape Oscillations and Spreading Dynamics of Picolitre Droplets Impacting on a Surface. Phys. Fluids 2023, 35, 122010 10.1063/5.0174511.

[ref53] Vaughn B. S.; Tracey P. J.; Trevitt A. J. Drop-on-Demand Microdroplet Generation: A Very Stable Platform for Single-Droplet Experimentation. RSC Adv. 2016, 6, 60215–60222. 10.1039/C6RA08472A.

[ref54] Van Berkel G. J.; Kertesz V.; Orcutt M.; Bentley A.; Glick J.; Flarakos J. Combined Falling Drop/Open Port Sampling Interface System for Automated Flow Injection Mass Spectrometry. Anal. Chem. 2017, 89, 12578–12586. 10.1021/acs.analchem.7b03899. 29112402

[ref55] Bhattacharyya I.; Maze J. T.; Ewing G. E.; Jarrold M. F. Charge Separation from the Bursting of Bubbles on Water. J. Phys. Chem. A 2011, 115, 5723–5728. 10.1021/jp102719s. 21090734

[ref56] Kebarle P.; Verkerk U. H. Electrospray: From Ions in Solution to Ions in the Gas Phase, What We Know Now. Mass Spectrom. Rev. 2009, 28, 898–917. 10.1002/mas.20247. 19551695

[ref57] Hsu C. Y.; Prabhu G. R. D.; Chang C. H.; Hsu P. C.; Buchowiecki K.; Urban P. L. Are Most Micrometer Droplets (>10 Μm) Wasted in Electrospray Ionization? An Insight from Real-Time High-Speed Imaging. Anal. Chem. 2023, 95, 14702–14709. 10.1021/acs.analchem.3c02799. 37725015

[ref58] Mortensen D. N.; Williams E. R. Ultrafast (1 Μs) Mixing and Fast Protein Folding in Nanodrops Monitored by Mass Spectrometry. J. Am. Chem. Soc. 2016, 138, 3453–3460. 10.1021/jacs.5b13081. 26902747

