# Supplementary material for: Rapid and Sensitive Chemical Analysis of Individual Picolitre Droplets by Mass Spectrometry
Source: Anal Chem. 2024 Dec 24;97(1):854–61. doi: 10.1021/acs.analchem.4c05458 (PMC11740182; doi:10.1021/acs.analchem.4c05458)
Supplement: Supplementary file 1 — ac4c05458_si_001.pdf [file ac4c05458_si_001.pdf]

Supporting Information

Rapid and Sensitive Chemical Analysis of Individual Picolitre Droplets by Mass Spectrometry

Jim S. Walker and Bryan R. Bzdek

School of Chemistry, University of Bristol, Cantock's Close, Bristol, BS8 1TS, UK

Table of Contents

Figure S1: 2

Figure S2: 3

Figure S3: 4

Figure S4: 5

Figure S5: 6

Figure S6: 7

Figure S7: 8

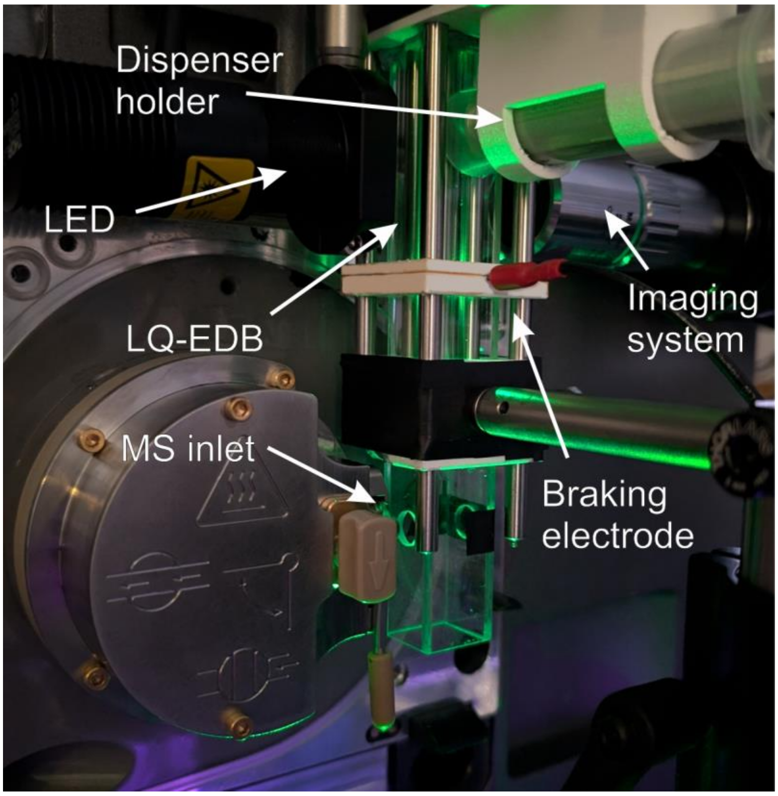

Figure S1: A photograph showing the experimental apparatus.

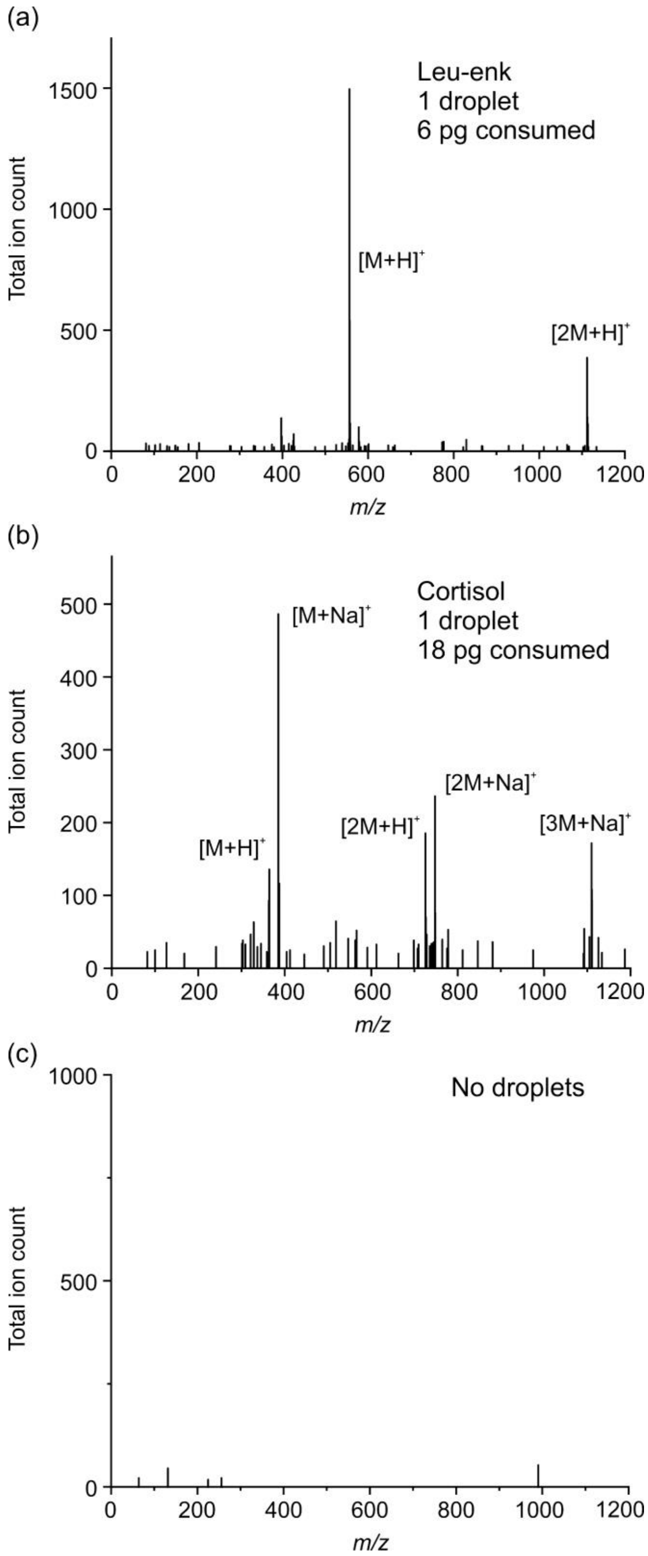

Figure S2: Example droplet mass spectra for individual aqueous picolitre droplets containing (a) leucine-enkephalin (50 μm diameter droplet, 180 μM + 0.1% formic acid) and (b) cortisol (58 μm diameter droplet, 500 μM). Panel (c) shows a typical background spectrum recorded between droplets.

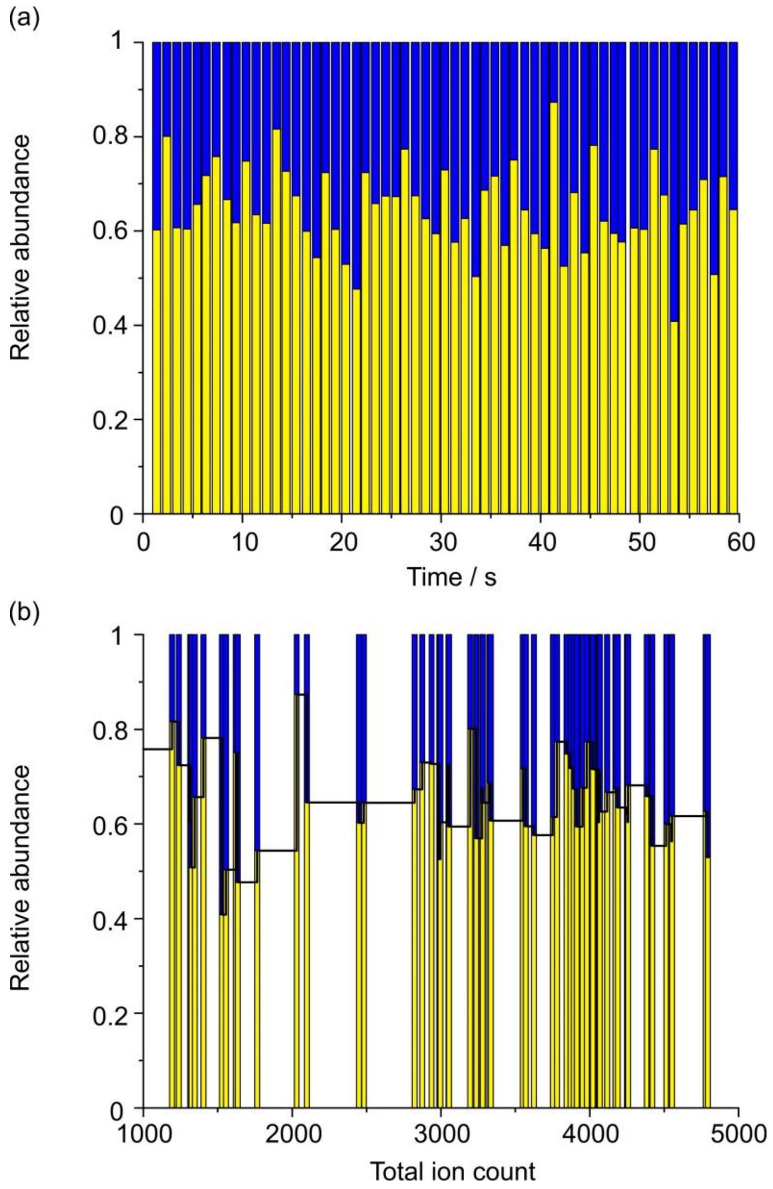

Figure S3: Relative abundance of the +1 (blue) and +2 (yellow) charge states for ~60 angiotensin II droplets as a function of (a) time and (b) total ion count. Droplets with lower total ion counts exhibit larger variations in detected charge states.

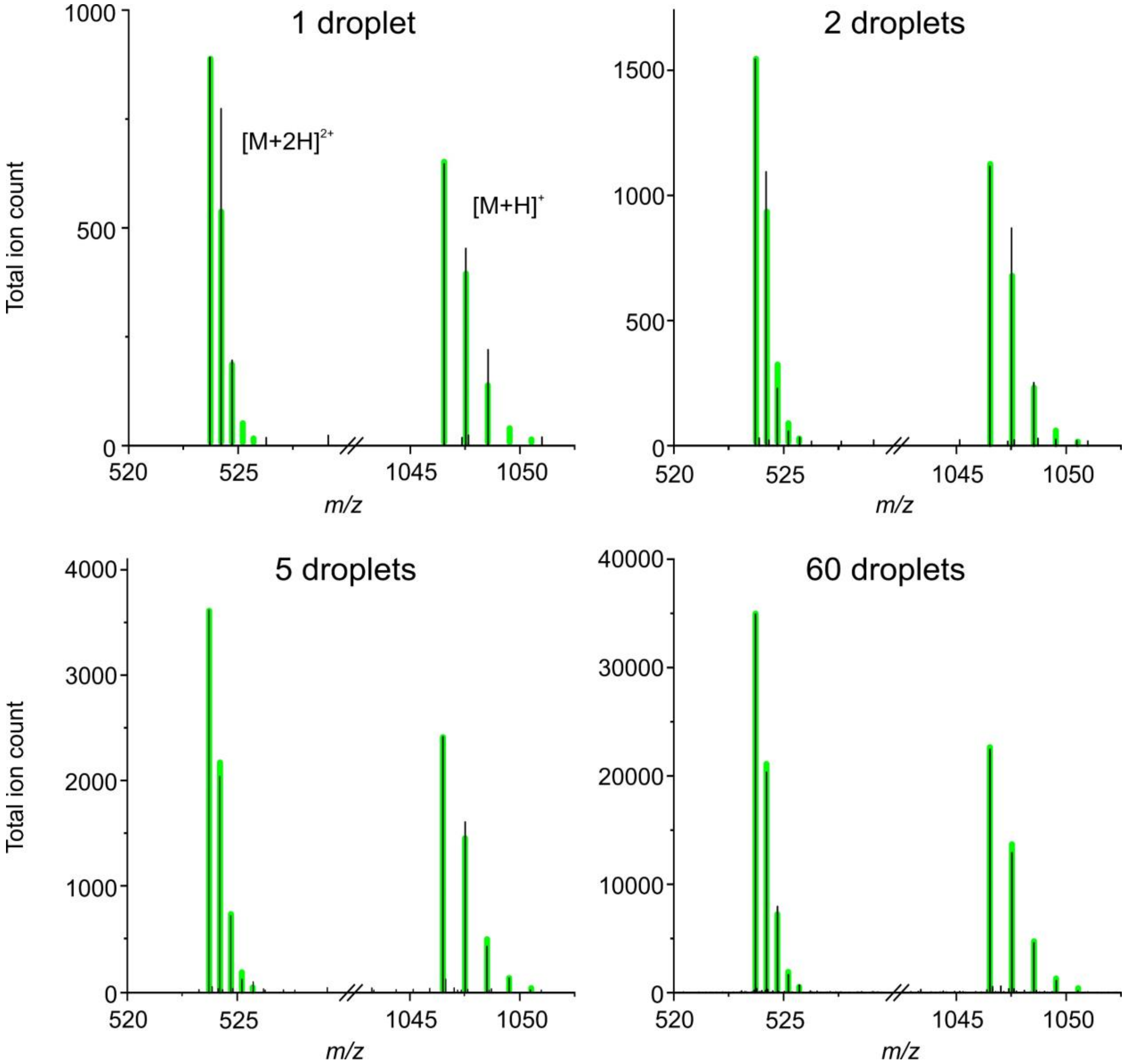

Figure S4: SDMS mass spectra showing the isotopic distributions for the +1 (1047 *m/z*) and +2 (524 *m/z*) angiotensin II molecular ions, integrated across (a) 1, (b) 2, (c) 5, and (d) 60 droplets. The theoretical isotopic abundance distribution is shown in green.

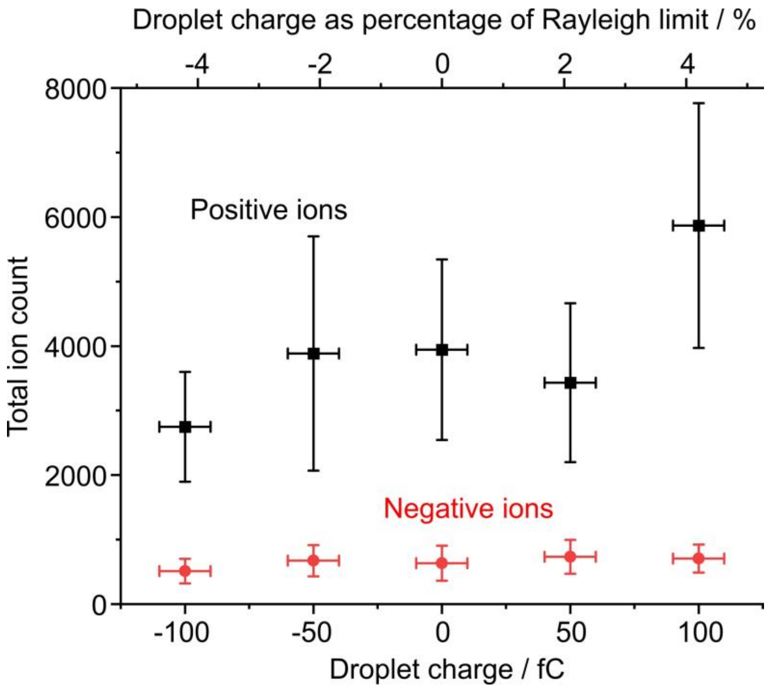

Figure S5: The effect of initial droplet charge on total ion count per droplet. Data are shown for both positive (black) and negative (red) analyte ions detected from individual 58 pL droplets containing 480 µM angiotensin II having different initial net charges.

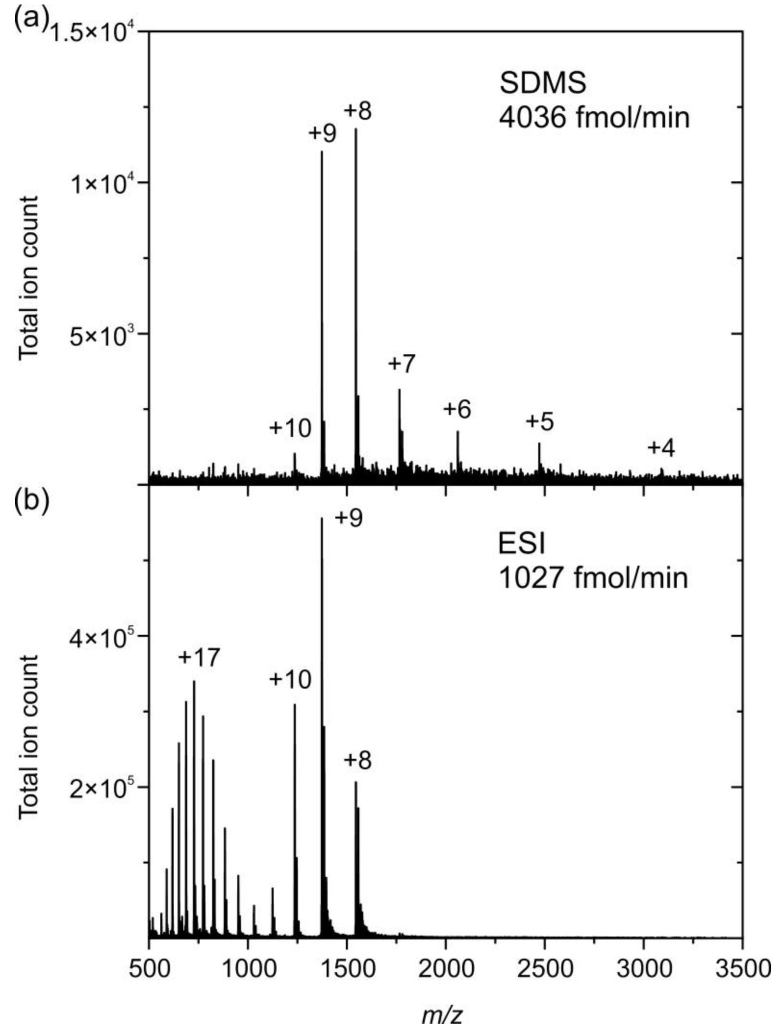

Figure S6: Example 10 s-integrated cytochrome-C mass spectra for (a) SDMS (10 μM in 0.1% formic acid) and (b) ESI (1 μM in 0.1% formic acid). The charge state distribution for the single droplet measurements is shifted to lower charge states compared to the ESI measurement. The weighted average charge states are +8.0 and +14.3 for SDMS and for ESI, respectively. The ion yields are $\sim 3\times 10^{-7}$ and $\sim 9\times 10^{-5}$ for SDMS and ESI, respectively.

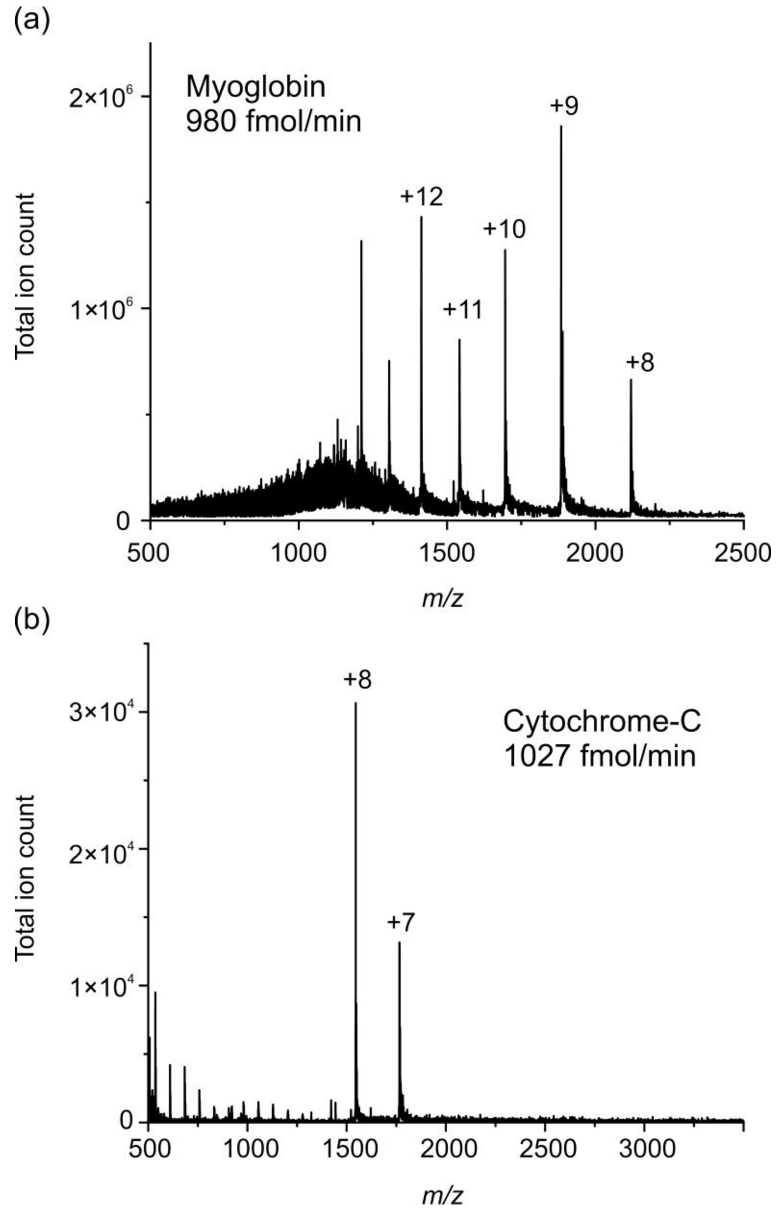

Figure S7: (a) ESI mass spectrum for myoglobin (1 μM) in 10 mM ammonium acetate buffer and 0.1% formic acid, 60 s integration. The weighted average charge state is +11.1, and the ion yield is ~5×10^{-5}, values similar to those measured by SDMS analysis of single picolitre droplets in 0.1% formic acid (Fig. 6a in the main text). The following settings were used for this ESI mass spectrum: capillary voltage = 3 kV, sampling cone voltage = 150 V, source offset voltage = 30 V. (b) ESI mass spectrum for cytochrome-c (1 μM) in 10 mM ammonium acetate buffer, 10 s integration. The weighted average charge state is +7.7, and the ion yield ~1×10^{-6}. The following settings were used for this ESI mass spectrum: capillary voltage = 1.5 kV, sampling cone voltage = 0 V, source offset voltage = 30 V.
